# Provider Perspectives on a Tobacco-Free Workplace Program in Healthcare Settings Serving Rural and Medically Underserved Areas of Texas: A Mixed Methods Study on Perceived Resource Availability and Value

**DOI:** 10.3390/ijerph23070898

**Published:** 2026-07-12

**Authors:** Abigail E. Bergey, Isabel Martinez Leal, Maggie Britton, Asfand B. Moosa, Tzuan A. Chen, Tarik D. Goulbourne, Ammar D. Siddiqi, Teresa Williams, Kathleen Casey, Lorraine R. Reitzel

**Affiliations:** 1 Department of Behavioral Science, The University of Texas MD Anderson Cancer Center, 1155 Pressler Street, Houston, TX 77030, USA; 2 Department of Psychological, Health, and Learning Sciences, University of Houston, McElhinney Hall, 3623 Cullen Blvd., Houston, TX 77204, USA; 3 Department of Health Initiatives, National Jewish Health, 1400 Jackson Street, Denver, CO 80206, USA; 4 HEALTH Research Institute, University of Houston, 4349 Martin Luther King Blvd., Houston, TX 77204, USA; 5 School of Medicine, University of California—San Francisco, 533 Parnassus Ave, San Francisco, CA 94143, USA; 6 Integral Care, 1430 Collier St., Austin, TX 78704, USA

**Keywords:** tobacco use, medically underserved areas, substance use treatment centers, medical healthcare centers, rural areas, health equity, tobacco-free workplace program, readiness for change, change valence, resource availability

## Abstract

**Highlights:**

**Public health relevance—How does this work relate to a public health issue?**

- Compared to the general population, smoking rates are at least twice as high among people with substance use disorders, a group that is overrepresented in rural/medically underserved areas in the United States.

**Public health significance—Why is this work of significance to public health?**

- Identifies barriers to and potential interventions for tobacco-free workplace programs as well as differences among healthcare settings that may contribute to successful future implementation outcomes.
- Outlines implications for strategic allocation of limited resources to organizations that will maximize impact.

**Public health implications—What are the key implications or messages for practitioners, policy makers and/or researchers in public health?**

- Providers at medical healthcare centers are significantly more likely to cite time as a resource barrier to tobacco cessation compared to those at substance use treatment centers; substance use treatment centers may be better positioned for early adoption of tobacco-free workplace programs.
- To strengthen organizational readiness and build greater capacity for tobacco cessation, provider attitudes, and organizational practices in treating tobacco dependence must be further developed and examined.

**Abstract:**

People with substance use disorders (SUDs) smoke at elevated rates compared to the general population, experience significant health disparities, and are overrepresented in rural and medically underserved areas with limited substance use treatment centers (SUTCs), leading individuals to seek care at medical healthcare centers (MHCs) (i.e., organizations providing comprehensive primary and preventive care). Thus, understanding MHCs’ and SUTCs’ capacity for adoption of tobacco-free workplace programs (TFWPs) that involve tobacco-free workplace policies and tobacco use treatment delivery is vital. Centers were continuously enrolled from August 2021 to January 2024, spanning the official duration and ending of the COVID-19 pandemic. As part of a mixed methods study, healthcare providers (*N* = 347) from 9 MHCs (*n* = 173) and 10 SUTCs (*n* = 174) in Texas were surveyed; 85 providers and clinical managers from a subset of this sample, drawn from 5 MHCs and 7 SUTCs, completed interviews about their perception of resource availability for, and value of, TFWPs. The organizational readiness for change theory guided the development of investigator-generated surveys and interviews focused on assessing the constructs of resource availability for and valuing of TFWPs. Quantitative findings generally revealed similar perceptions on value and resource availability. However, more MHC than SUTC providers endorsed lack of time as a barrier to delivering cessation care (*p* = 0.006). Qualitative themes included lack of training and resources to build capacity; limited institutional frameworks, policies, and practices; underprioritizing of tobacco cessation treatment; and erroneous assumptions that tobacco use sustains SUD sobriety. Results emphasize that MHCs need shorter intervention strategies, while SUTCs may be poised for longer interventions as supported through greater endorsement by MHC compared to SUTC providers of time as a barrier to deliver cessation care alongside similar perceptions on value and resource availability across both settings (MHCs, SUTCs). Overall, findings reinforce the need to build greater capacity across healthcare settings for patients’ tobacco use treatment.

## 1. Introduction

Tobacco use is the leading cause of preventable death and disability in the United States (US) [1]. Nearly one in five of all annual deaths in the US is linked to cigarette smoking (the most common form of tobacco use) and exposure to secondhand smoke [2]. Although the overall rate of cigarette smoking among US adults has declined in recent decades, currently estimated at 11.6% [3], tobacco use remains disproportionately concentrated among people who have been socially, economically, and/or historically marginalized. For example, people with substance use disorders (SUDs) smoke cigarettes at rates at least twice as high as those in the general population, with some subgroups (e.g., those with opioid use disorders, stimulant use disorders) reaching prevalence rates over 65% [2,4,5,6,7]. This enduring disparity underscores tobacco use as both a public health concern and social justice issue contributing to health inequities [8,9]. Specifically, tobacco-related mortality rates are higher among people with SUDs compared to the general population [10]. Geographic differences further compound these disparities [11,12], as those living in rural and medically underserved areas (MUAs) have higher rates of tobacco use [13,14], are less likely to attempt quitting [15,16], and experience greater tobacco-related mortality compared to those in urban or higher-resourced areas [17].

Social determinants of health provide an important contextual lens through which to understand persistent tobacco-related inequities, particularly in rural and MUAs. Structural factors account for up to 50% of county-level variation in health outcomes across the US [18], directly impacting people’s ability to access tobacco use treatment. For example, geographic barriers to care, such as limited transportation [19] and long travel distances, are associated with reduced access to specialized medical care among people living in rural areas as opposed to urban settings [11,12]. These structural inequities are reflected in national-level quit ratio trends from 2010 to 2020, which reveal that although the likelihood of quitting smoking increased over time for both rural and urban populations, the rate of improvement has been slower in rural areas, widening the gap over time [20]. This is particularly concerning for people with SUDs living in rural and MUAs, who experience lower rates of treatment initiation and continuity compared to those living in urban settings [21,22].

Public health efforts have historically played a critical role in reducing tobacco use through a range of population-level interventions, including smoke-free air laws, increased tobacco taxation, and mass media campaigns [13,23,24,25,26,27]. While these strategies have contributed to significant increases in quit attempts made and declines in smoking prevalence over time, disparities persist in equitable adoption and implementation of tobacco control policies and programs, particularly across geographic and socioeconomic lines [24]. Workplaces in urban areas, for instance, are more likely to institute smoke-free policies [14,28,29], whereas rural areas often lack resources (e.g., for addressing tobacco dependence) and political support (e.g., mandates by local government) necessary to achieve tobacco-free environments [13,30]. To address these enduring gaps and to reduce tobacco-related disparities among people with SUDs, there is a pressing need to strengthen tobacco control capacity within substance use treatment settings serving rural and MUAs [31]. This includes expanding access to tobacco dependence treatment resources and building the systems and workforce needed to implement evidence-based tobacco control strategies.

Unfortunately, rural and MUAs face a significant shortage of substance use treatment centers (SUTCs), prompting a call to integrate behavioral health (i.e., SUDs and mental health conditions) services within medical healthcare centers (MHCs), such as rural healthcare clinics and Federally Qualified Health Centers (FQHCs) [32]. Therefore, advancing tobacco management efforts within the diverse settings where people with SUDs receive care is critical. One effective approach involves implementing comprehensive, evidence-based tobacco-free workplace programs (TFWPs) that integrate the development of tobacco-free policies, training in tobacco treatment, and the provision of tobacco cessation services to create supportive environments and systems that enhance the likelihood of successful cessation [33]. However, there are numerous barriers, including time constraints, high turnover rates, and staff perceptions, to tobacco control efforts in these settings [34,35,36,37,38,39,40,41], as well as limited funding available to support such efforts [42]. Thus, it is essential to allocate what limited resources are available to organizations with the greatest potential for successful implementation [43].

Two complementary theoretical frameworks shed light on organizational factors influencing successful adoption and implementation of TFWPs: diffusion of innovations theory and organizational readiness for change theory. Diffusion of innovations theory highlights predictors of adoption, such as the compatibility of the TFWP with the setting’s values, norms, and perceived needs, helping identify early program adopters [43]. Organizational readiness for change theory focuses on the internal conditions that determine whether the organization is prepared to implement such a change successfully. Organizational readiness for change theory has been widely applied in health system research to identify determinants of successful implementation [44,45]. Two central determinants include informational assessment (i.e., whether necessary resources are available to support the change), a function of change efficacy, and change valence (i.e., the extent to which people value the proposed change) [46]. Given the growing emphasis on integrating behavioral health into MHCs to address SUD-related disparities in rural and MUAs, it is important to evaluate whether MHCs and SUTCs in these areas are prepared to successfully adopt TFWPs. To date, there has been limited work examining whether MHCs and SUTCs differ in terms of resource availability for and perceived value of TFWPs. However, prior studies offer some indication that such differences may be present.

Numerous resource limitations have been identified as persistent barriers to the provision of tobacco use treatment services and adoption of tobacco-free policies [47,48,49] in behavioral health settings, including insufficient time and a lack of accessible community resources [41]. Lack of resources is one of the main barriers to implementation of tobacco-free policies and tobacco treatment services cited by SUD treatment program directors [48]. Similarly, a study of FQHCs found that only 50% of centers had access to two or more tobacco treatment resources (e.g., fax referral to a state Quitline, group tobacco cessation support, individual tobacco cessation support at the clinic), while the remaining FQHCs were evenly split between having only one resource or none at all (25% each) [50].

TFWPs may be less valued in SUTCs, potentially due to SUTC provider perceptions regarding the appropriateness of treating tobacco use alongside non-nicotine SUDs. Historically, providers in these settings have often been unaware that treating tobacco use alongside other SUDs enhances the likelihood of successful recovery. They may also underestimate patients’ interest in quitting (i.e., found to range from moderate consideration to serious consideration [51]) and their capacity to do so [52]. Additionally, many SUTCs have maintained a smoking-permissive culture, which can further diminish support for tobacco use treatment [53]. In contrast, MHCs may be less influenced by such norms due to a more whole-health approach [54] and may hold less entrenched views about changing tobacco use behaviors in this population that subvert program adoption. Furthermore, TFWPs’ valuation may differ in SUTCs compared with MHCs due to lower SUTC staff investment in intervention practices [55]. Provider perceptions of [40], and staff/leadership investment in, implementation of TFWPs in SUTCs serving patients with SUDs are important factors affecting program implementation (e.g., staff overestimate coworker implementation concerns) [56]. Patients in SUTCs may be motivated to change their tobacco use behaviors but fail to receive support from SUTC healthcare providers [57,58].

This study contributes to existing research by providing a mixed methods perspective on provider perceptions of resource availability for and change valence related to adoption of a TFWP in MHCs and SUTCs. By comparing and relating results from both quantitative and qualitative data perspectives, this study benefits from a more comprehensive understanding of organizations’ capacity to adopt a TFWP in these settings than by using either method alone.

## 2. Materials and Methods

### 2.1. Study Design

**Figure 1:**
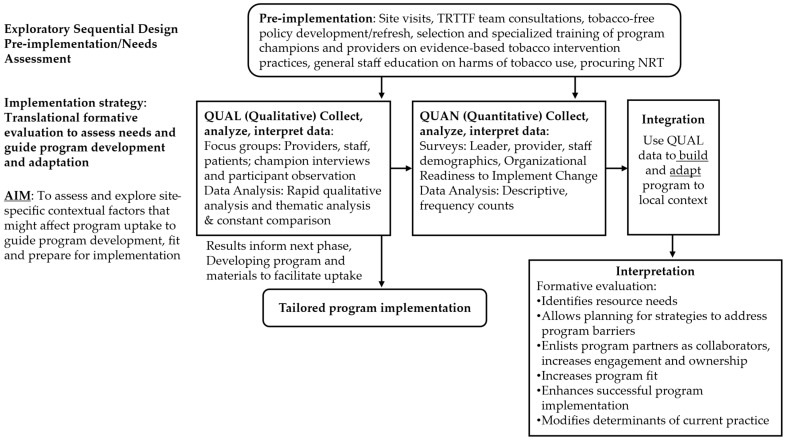
Pre-Implementation Mixed Methods Framework for Implementing a Tobacco-Free Workplace Program in Rural Texas. Note: TRTTF = Taking Rural Texas Tobacco Free; NRT = nicotine replacement therapy.

This study reports on the quantitative and qualitative findings from Phase 1 of a multistage mixed methods evaluation design consisting of three phases: pre-implementation, implementation, and post-implementation [59]. Each phase had a different research aim and core mixed methods design. Phase 1 was based on an exploratory, sequential design and was the pre-implementation, formative evaluation stage (see Figure 1).

This phase entailed a needs assessment/formative evaluation approach focused on assessing community partners’ needs as well as potential barriers and assets regarding implementation of an evidence-based TFWP. The aim guiding this phase was identification of site-specific contextual factors that might affect program adoption, to inform tailoring the program to local settings, thus laying the groundwork for effective implementation. Quantitative data collection consisted of surveys of leadership and staff that assessed current tobacco-free workplace policies and practices, including screenings for tobacco use, as well as any tobacco treatment services provided, and beliefs about TFWPs. Qualitative data consisted of individual or group interviews with staff (healthcare providers and clinical managers) focused on attitudes about providing tobacco treatment, beliefs about patient interest and capacity to change tobacco use, as well as any potential barriers or facilitators to program implementation (i.e., contextual factors that could affect implementation). Group interviews are synonymous with focus groups [60]. The quantitative and qualitative components were conducted independently, with respective analysts blinded to the results of the other dataset until the final analysis, when data were merged through comparison and synthesis [61]. The purpose guiding the adoption of a mixed methods design in this study was complementarity, in which qualitative findings served to enhance and elaborate upon quantitative results [62]. The combining of the findings from the two components serves to overcome their respective weaknesses, thus extending the capability of each, yielding a more complete understanding of the implementation context than either method in isolation [63].

### 2.2. Procedures and Participants

#### 2.2.1. Eligibility and Recruitment

This study reports on an iteration of Taking Texas Tobacco Free (TTTF) [64], a comprehensive TFWP designed to support community and healthcare organizations in building capacity for policies and practices that reduce tobacco use and secondhand smoke exposure among staff, patients, and visitors. The term “comprehensive” is used here to refer to a multi-component tobacco control program combining three evidence-based practices, including the adoption of a comprehensive or total tobacco-free workplace policy, which covers all tobacco products, combustible, non-combustible and electronic, throughout participating healthcare center grounds; training of staff on treating tobacco use; and delivery of regular tobacco use assessments and interventions. Since its launch in 2013, TTTF has evolved to address the needs of diverse healthcare settings across Texas. The current iteration, titled Taking Rural Texas Tobacco Free (TRTTF), specifically targeted MHCs and SUTCs located in and/or serving populations from rural counties and/or MUAs, reflecting a strategic focus on reducing tobacco-related disparities in these high-need areas. To identify potential partner centers, the project team assembled a comprehensive list of healthcare centers across Texas, specifically targeting MHCs and SUTCs in rural and/or MUAs. Eligible partners included MHCs and SUTCs whose leadership self-identified their organizations as meeting the geographic criteria and expressed a commitment to enhancing their tobacco control efforts, including screening and cessation services.

The recruitment list was developed using a combination of online searches, professional organization directories, and prior collaborations. Recruitment efforts primarily involved direct email outreach, which included a one-page flyer describing the TRTTF initiative. Additional outreach strategies included participation in professional conferences and meetings relevant to rural healthcare stakeholders (e.g., Texas Association of Rural Health Clinics [65], Texas Association of Community Health Centers [66], Texas Association of Addiction Professionals [67]). At these events, the program was promoted via exhibitor booths staffed by team members, distribution of flyers in attendee bags, and/or oral presentations. Interested parties could share their contact details with the study team or complete an online Qualtrics (https://www.qualtrics.com/) survey indicating whether their center met the rural or MUA criteria.

#### 2.2.2. Study Procedures

A total of 28 centers (10 MHCs and 18 SUTCs) were eligible to participate in this study. Out of these, 19 centers (9 MHCs and 10 SUTCs) were enrolled on a rolling basis in the program between August 2021 and January 2024. Due to a transition in university affiliation for several members of the project team, the work was paused for three months while the grant was transferred. During this period, there was no interaction with partners. This hiatus caused considerable delays in data collection, requiring a re-engagement process with enrolled partners. Before program implementation began, a baseline (“pre-implementation”) anonymous survey was administered to center staff and providers. The survey was preceded by a cover letter detailing the study’s purpose and informed consent information. At the conclusion of the letter, staff were asked to indicate their “consent to participate.” Center leadership or a designated Program Champion (a staff member appointed to liaise with the study team, who was not financially compensated for this role) distributed the survey link to staff via email. Survey data collection occurred between September 2021 and February 2024 via an electronic Qualtrics survey distributed to participating healthcare centers. Our data collection period occurred during the ongoing COVID-19 pandemic, putting a burden on data collection due to center closure, suspension of services, staff shortages, and center attrition.

The surveys (see File S1: Leader Readiness; File S2: Pre-Implementation Survey) collected information on center affiliation, asked respondents to identify themselves as either general staff or healthcare providers, and included questions across topic areas relevant to program implementation, such as baseline status of a center’s tobacco-free workplace policy, and current tobacco screening and care practices. Healthcare providers were defined as individuals holding relevant clinical or support credentials (e.g., nurse practitioner, licensed vocational nurse, medical assistant, licensed chemical dependency counselor, peer support specialist, etc.). General staff included employees who did not provide direct services to clients such as administrators, front-office workers, receptionists, IT personnel, etc. Respondents were informed that they could receive a $10 Amazon e-gift card for survey completion and were directed to a separate form to provide contact information for gift card delivery. No other identifying information was collected.

Select staff were invited to participate in virtual individual or group interviews (see File S3: Pre-Staff Interview Guide) centered on understanding barriers and facilitators to tobacco treatment practices, unique center characteristics, attitudes towards treating tobacco dependence, and perceptions of the importance of tobacco-cessation treatment in healthcare organizations from providers and clinical managers. Interviews were usually conducted following staff training, which included information about the TRTTF program; a third of the participating centers scheduled the interview just prior to a staff training on the harms of tobacco use and evidence-based tobacco treatments. Participants were selected for the qualitative procedures via criterion sampling, a type of purposeful sampling in which participants are selected based on predetermined criteria. In this case, to ensure that interview participants (i.e., staff: providers or clinical managers) would be knowledgeable about current tobacco use policies and procedures at their respective centers [68]. Prior to the interview, staff were read aloud a cover letter detailing the program’s purpose and informed consent information. Following oral consent, these approximately 25–50 min interviews (depending on the number of participants, i.e., individual or group interviews) were audio and video recorded. Staff were compensated with a $20 e-gift card for their participation. Interviews took place virtually from October 2021 to April 2025 and each was conducted by an anthropologist and public health researcher (I.M.L.).

#### 2.2.3. Approvals

The study was initially approved by the Institutional Review Board (IRB) at the University of Houston (STUDY00002885, initial approval 20 April 2021). Upon transition of much of the project team to The University of Texas MD Anderson Cancer Center (MD Anderson), subsequent approval was then granted by the Quality Improvement Assessment Board (QIAB; study ID 930, initial approval 21 November 2022) at this institution and work with partners resumed. MD Anderson determined that the project did not meet the regulatory definition of human subjects’ research and thus did not require IRB review, but rather required QIAB approval, as it was focused on quality improvement in healthcare.

#### 2.2.4. Quantitative Sample

In total, 542 staff from MHCs (*n* = 9) and 490 from SUTCs (*n* = 10) responded to the pre-implementation survey, representing average response rates of 73.61% and 74.63%, respectively, based on leadership-reported staff census. For analytic purposes, the sample was refined to include only healthcare providers, 173 from MHCs and 174 from SUTCs, since only these individuals were asked about barriers to providing tobacco treatment. This subset reflected response rates of 49.19% for MHC providers and 49.45% for SUTC providers.

#### 2.2.5. Qualitative Sample

The qualitative sample included representatives from 12 healthcare centers: 5 MHCs and 7 SUTCs. These centers represent a subset of the quantitative sample that completed pre-implementation surveys; the remaining 7 centers (*n* = 4 MHCs and *n* = 3 SUTCs) withdrew from the study prior to completing this phase. A total of 70 providers and 15 clinical managers participated in 16 virtual semi-structured individual and group interviews (consisting of 4–10 participants) conducted pre-program implementation. The rationale for conducting individual vs. group interviews was to accommodate partner preference. For smaller centers, pulling a lot of personnel out of service at one time for a group interview was not feasible, so individual interviews were conducted.

### 2.3. Quantitative Measures

#### 2.3.1. Healthcare Center Characteristics

Characteristics of healthcare centers were provided by center leadership regarding the number of counties served, unique patients served annually, total patient visits annually, staff, healthcare providers, as well as the presence of a comprehensive tobacco-free workplace policy (yes or no).

#### 2.3.2. Perceptions of Resource Availability

Providers responded to six face-valid survey items assessing their perceptions of organizational resources and barriers related to tobacco-free workplace policy enforcement and tobacco treatment delivery. The first item assessed respondents’ endorsement of, or agreement with, a tobacco-free workplace policy-related concern: “My center lacks the resources to adequately enforce the policy,” with response options of yes (endorsed) or no (not endorsed).

The other five items assessed perceived barriers related to the center’s capacity to provide smoking or tobacco use cessation counseling, including: time (i.e., “Lack of time for healthcare providers to provide tobacco cessation services”), reimbursement/cost (i.e., “Lack of reimbursement/it costs too much to do this”), community resources (i.e., “Lack of community resources to refer patients”), patient education material (i.e., “Lack of patient education material”), and training (i.e., “Lack of training”). Respondents were asked to indicate on a 5-point Likert scale from “not at all important” to “extremely important” how important each barrier was at their center. For analysis, responses were dichotomized as yes (extremely important or very important) or no (moderately important, slightly important, or not at all important).

#### 2.3.3. Perceived Value

Providers responded to five face-valid survey items assessing their perceived value of tobacco-free workplace policies and tobacco treatment. The first three items asked respondents to indicate agreement with a tobacco-free workplace policy-related concern: “Tobacco cessation counseling is an important part of my center’s mission”; “Tobacco free workplace policies are important because they provide a clean and safe environment for our staff to work in and patients to receive care in”; and “Tobacco free workplace policies may help our patients and staff quit smoking.” Respondents indicated on a 5-point Likert scale ranging from “strongly disagree” to “strongly agree.” For analysis, participant responses were dichotomized as yes (strongly agree or agree) or no (neither agree nor disagree, disagree, or strongly disagree). The last two items assessed perceived barriers to the center’s capacity to provide smoking or tobacco use cessation counseling, including: leadership investment/interest (i.e., “Lack of center leadership investment”) and staff investment/interest (i.e., “Lack of staff investment”). Each item was rated on a 5-point Likert scale ranging from “not at all important” to “extremely important.” For analysis, responses were dichotomized as yes (extremely important or very important) or no (moderately important, slightly important, or not at all important).

### 2.4. Data Analysis

#### 2.4.1. Quantitative Data

Descriptive statistics summarized key study variables. Generalized linear mixed models tested for differences in provider responses to resource availability items and perceived value items for policy and tobacco treatment provision by healthcare center type, controlling for an extant tobacco-free workplace policy. Due to the nested structure of data and missing responses on a non-mandatory survey question, the analytic sample size varied slightly across models. The total analytic sample was 347 providers. All analyses were conducted using SAS version 9.4, with significance at *p* < 0.05.

#### 2.4.2. Qualitative Data

A professional transcription service that was HIPAA-compliant transcribed the interviews verbatim. Interviews were organized in a Microsoft Teams drive to facilitate coordination of data analysis. A rapid qualitative analysis (RQA) approach [69] was used in conjunction with matrix displays [70] to facilitate timely interpretation of findings to tailor program development and implementation. RQA was specifically selected because this streamlined approach was developed for use in health services and implementation science, where actionable findings are needed in shorter timeframes to inform implementation processes in close to real-time. Our research questions, along with the organizational readiness for change theory [46], guided qualitative processes from data collection—writing up interview guides and sampling strategies—to data analysis, selection of analytic methods, developing summary templates and matrices, and final writing up of findings. Research questions and templates were drawn up by I.M.L, a cultural anthropologist and public health researcher trained in qualitative research methods based on prior experience in the implementation of tobacco management interventions among various marginalized and minoritized groups, and field tested and refined based on participant responses. Study team members reviewed data collection and analytic instruments. Structured templates were drawn up, consisting of key thematic domains based on the content of the interview guides, to organize and summarize data [71]. Use of deductive, predetermined domains was beneficial in streamlining and directing the process of analysis [72]. This method produced concise summaries consisting of two–three-page bulleted entries using Microsoft Word, and exemplary quotes that facilitated organization of qualitative data into structured matrices, also drawn from research and interview questions. Matrices were organized using Microsoft Excel. The addition of a domain of “other observations” was also created to capture any topics not encompassed by existing domains, ensuring that the analysis remained open and flexible during the analytic process.

Two qualified analysts, (I.M.L.) and (A.E.B.), a research assistant (a graduate student in counseling psychology with RQA and qualitative research training), independently summarized the same five transcripts to establish consistency in the understanding and application of domains. All RQA summaries included links to the transcripts for access to the raw data. The two analysts met to confirm the accuracy of the domains and discuss any discrepancies; remaining transcripts were independently summarized and discussed using this same process of meeting to ensure agreement. The last stage of analysis entailed using thematic analysis [73] to constantly compare matrix entries to identify patterns and salient themes. This was an inductive process, in which responses within and across transcripts were compared and synthesized across domains to develop themes during final analysis. Once the RQA summaries had been completed, they were sent to the implementation team members (i.e., health education specialists) who liaised with TRTTF partners to review and translate findings into action items for adapting interventions to local settings. Action items were added to bi-weekly team meetings for discussion and further refinement and to note progress on completion of recommended tasks. Health education specialists discussed and coordinated with program champions on the feasibility of and processes for implementing these steps to tailor the program to center workflows and patients’ needs. The timeline for conducting, transcribing, and summarizing interviews, and loading summaries into matrices was generally two weeks per interview. The process for team analysis and discussion data, sharing of findings with health education specialists, and writing up of action items for presentation to the study team during bi-weekly meetings took ~one to two months. To ensure methodological accuracy, conducting RQA analysts were guided by Kowalski et al.’s framework for planning and assessing rigor in rapid qualitative analysis (PARRQA) [74].

## 3. Results

The current study reports on findings from a mixed methods study on provider perceptions of resource availability for and change valence related to adoption of a TFWP in MHCs and SUTCs.

### 3.1. Healthcare Center Characteristics

Table 1 includes a summary of the characteristics of the healthcare centers that agreed to participate in the TFWP, as reported by their leadership at pre-implementation. There were no significant differences between MHCs and SUTCs on these or other demographic variables (see Table 1).

### 3.2. Quantitative Results

Generalized linear mixed models tested for differences in provider responses by center type; nested data structure was considered, and tobacco-free workplace policy existence was controlled. Table 2 and Table 3 provide information on differences in provider perceptions for informational assessment and change valence, respectively.

#### 3.2.1. Information Assessment

Analyses tested for differences in provider responses by center type according to provider perceptions of existing resources to enforce TFWP and resource barriers to cessation care practices. Analyses revealed that providers at MHCs, in comparison to SUTCs, perceive lack of time to deliver care to be a greater resource barrier.

#### 3.2.2. Change Valence

Findings on differences between MHCs and SUTCs according to provider perceived value of a tobacco-free workplace policy and tobacco treatment across center types indicate comparable provider valuation of TFWP and tobacco treatment in both healthcare settings.

### 3.3. Qualitative Results

Qualitative analysis of interview transcripts yielded four main themes regarding provider perceptions of resource availability for and change valence related to adoption of a TFWP (Table 4): (1) Efficacy Gaps—additional training and resources are needed to build capacity, (2) Organizational Factors—limited institutional frameworks, policies, and practices, (3) Valuing—tobacco dependence care was valued as compatible but not a treatment priority, and (4) Conflicting Attitudes—negative assumptions about patient tobacco use and smoking to maintain sobriety. Each of the four themes is further described below, situated in the context of quotes to provide evidence of findings.

#### 3.3.1. Efficacy Gaps—Additional Training and Resources Are Needed to Build Capacity

Providers from MHCs and SUTCs reported efficacy gaps in their ability to address tobacco use. Specifically, a lack of training and resources emerged as two salient barriers to effective tobacco treatment service integration. Participants reported a need for further training to better provide services to their patients:

*“…we’ve been pretty successful with our employees but not our patients… So, I think we need to put a little more—emphasize it a little more. I think maybe with the proper training.”*

*(Provider, MHC #4)*

This emphasis on seeking better service provision is similarly illustrated through providers looking for more tools and resources to share with patients to expand their toolkits to help patients quit:

*“…because we don’t have the proper tools and resources to help them, the only thing we have now is the Quit Now number. We don’t really offer like, ‘Are you wanting to quit?’ We don’t offer that until we get the resources with you all. That’s when we’ll start offering, like, ‘Hey, are you wanting to quit?’”*

*(Provider, SUTC #6)*

*“I think we just have always needed more resources or more information for the patients, and us telling the patient that we are a smoke-free campus and that we have options to help them quit. If you don’t have information to hand to them or the product to hand to them, it definitely hurts their ability to be successful. So, I think now that we have all that, we will be better off.”*

*(Provider, SUTC #7)*

#### 3.3.2. Organizational Factors—Limited Institutional Frameworks, Policies, and Practices

Organizational factors like tobacco-free workplace policies, as well as current practices and frameworks related to addressing tobacco use, were limited among program participants. Organizational gaps primarily focused on lack of policies and available treatment services. Regarding tobacco-free workplace policies, many providers reported general lack of knowledge or absence of comprehensive policies. Most centers’ policy regarding tobacco use simply defaulted to state-mandated tobacco-free workplace policies:

*“I don’t think we have any policies about that [on-site tobacco use], but then I don’t think a lot of employees do smoke. I’m not too sure, honestly.”*

*(Provider, MHC #1)*

Some providers reported valuing the implementation of a tobacco-free workplace policy to assist with their own ongoing and past attempts to quit smoking:

*“In my experience, it’s so hard to kick. I think it’s the worst. It’s the nastiest, most expensive habit you can imagine, but I myself haven’t been able to stop smoking… Quite frankly, I’m not one to smoke in front of my employer’s building and stuff, but I don’t see many smokers around here, and that’s how I smoke… Yes, I can definitely learn a lot. I know it’s so hard [to quit].”*

*(Provider, SUTC #3)*

Additionally, providers shared their current tobacco treatment service provision. Providers reported sharing tobacco cessation information with patients, but a lack of access to providing further services:

*“I try to print some handouts, and I leave them out there on the table, but pretty much that’s all I do. We don’t have access to give them anything.”*

*(Provider, SUTC #5)*

Some providers identified a broader need for services and were unaware of available federal and state resources (e.g., Quitlines):

*“Maybe if there’s like an alternative that I can tell them, ‘There’s a number you call to speak about it whenever you decide if you want to stop,’ because sometimes, they don’t want to talk about it in person because I guess they feel like maybe it’s like an intervention almost.”*

*(Provider, MHC #5)*

#### 3.3.3. Valuing—Tobacco Cessation Was Valued as Compatible but Not a Treatment Priority

Providers generally valued tobacco treatment as compatible with their center’s mission and programming. However, many providers reported they felt addressing tobacco use was not a treatment priority. This was particularly prevalent in SUTCs; providers indicated that their treatment focus was on addressing other drugs:

*“Typically, they’ve gotten to the point where they’re willing to give up their drug of choice, whatever it is, but—and so that’s the main area of focus. I know and I agree as far as nicotine being a drug, and not helping with healing, but I think it is treated differently in the sense that we’re like, ‘Okay, you’re still smoking in the program, but obviously, you’re not using these other drugs,’ if that makes sense.”*

*(Provider, SUTC #2)*

*“I would say not the top priority. If they’re coming in with no alcoholism or—it’s not their number [one] priority at this time. It hasn’t been traditionally. It’s whatever the other person [‘s] substance of choice is.”*

*(Provider, SUTC #2)*

Some providers conceptualized tobacco as a better alternative for their patients and described encouraging tobacco use when the alternative choice involved other drugs (e.g., meth):

*“Yes, unfortunately a lot of the time, it is letting them embrace the lesser of two evils view because yes, if the choice is doing meth or smoking cigarettes like, ‘Hey, go buy a pack. That’s way better.’ So, that’s partly might be why is just because like [name] was saying, our population, they are coming from usually a much more serious situation, so stepping down to smoking is usually like a win for us.”*

*(Provider, SUTC #3)*

In MHCs, time was presented by providers as a barrier to addressing tobacco use. Attending to patients’ central concerns and treatment diagnoses took precedence for providers in these settings due to perceived limitations surrounding time:

*“I’m so busy. I’m going to address what they’re there for. Those extra things that I don’t hit on them… it says twenty minutes. By the time I actually get in there, I might have five, depending on if the patient was late or what’s going on. I don’t have a lot of time with them. The time I do have, I hit on the main points.”*

*(Provider, MHC #2)*

#### 3.3.4. Conflicting Attitudes—Negative Assumptions About Patient Tobacco Use and Smoking to Maintain Sobriety

Some providers perceived low willingness from patients regarding addressing their tobacco use; in direct refutation of their own report on patients’ expressed interest in quitting:

*“I would say 10% might be willing out of 90%, but I really was kind of shocked when people came in that some expressed that they wanted to quit smoking, to be honest with you.”*

*(Provider, SUTC #6)*

Providers reported anticipating conflict and anger from patients when bringing up tobacco use to patients who smoke. Some providers appeared to infer from one experience a presumption that older patients who smoke are unwilling to discuss changing their tobacco use behavior:

*“I feel like if I were to [bring up smoking] like for the older generation, for example, I’ve had a patient where they’re smokers for 20-plus years, and I asked them a question of like, ‘Have you thought of like stopping smoking?’ They get a little bit angry. They don’t really like having that question being asked to them.”*

*(Provider, MHC #5)*

At SUTCs, providers shared concerns about disruption of patient sobriety (from other non-nicotine substances) when quitting tobacco:

*“Then, I think also sometimes it is used as a tool for maintaining sobriety. So, someone might be using tobacco to stay away from meth, heroin, or whatever it was they were using before. So, they’re using that to help maintain sobriety.”*

*(Provider, SUTC #4)*

### 3.4. Integrated Mixed Methods Findings

Quantitative data from surveys and qualitative data from staff group and individual interviews were compared and merged to understand how each dataset contributes to advancing program implementation by tailoring the program to the needs of program partners. Table 5 presents a summary of the findings from the different components, merging and connecting them to provide insight into how data from both strands can inform the development of strategies and practical adaptations to fill identified resource gaps and address implementation barriers, thus facilitating program implementation.

## 4. Discussion

This study utilized a mixed methods approach to assess provider perceptions of resource availability for and valuation of a TFWP in MHCs compared to SUTCs prior to program adoption to better understand contextual factors that can influence implementation and thus advance organizational preparedness. The concept of “organizational readiness” described in the organizational readiness for change theory was an effective implementation theory to frame the analysis of factors that can enhance or impede effective program implementation [46]. This theory served to identify the degree to which determinants of successful implementation—availability of resources for and valuing of the TFWP—were present or absent in participating centers pre-implementation. The project team and program partners were thus able to coordinate making any necessary adjustments prior to implementation to support successful adoption. The quantitative portion of this study specifically investigated differences in provider perceptions of existing resources to enforce a tobacco-free workplace policy and to deliver tobacco use care in Texas MHCs and SUTCs serving rural/or medically underserved patients as well as their valuation of these evidence-based interventions. Past research on implementation of a TFWP in Texas suggests organizational demographics and readiness for change by implementing a TFWP may moderate changes in provider intervention delivery within behavioral health clinics [80]. Identifying organizational factors contributing to provider intervention delivery requires refined understanding of provider beliefs and perceptions involved in the TFWP adoption to facilitate program uptake [81].

Quantitative findings showed that providers in both healthcare settings generally valued tobacco-free workplace policies for promoting a clean, safe environment and supporting tobacco cessation, though with only about 60–70% of providers agreeing, indicating there is room for improvement. Across MHCs and SUTCs, providers also reported similarly low staff and leadership investment in tobacco treatment delivery and its relevance to their centers’ mission, suggesting comparable foundations for TFWP implementation. Providers in both settings endorsed adequate organizational resources to adopt tobacco-free workplace policies and reported similar gaps in resources needed to deliver tobacco use interventions, including a lack of training, reimbursement, referral resources, and patient education materials. One key difference emerged: providers at MHCs were significantly more likely than those at SUTCs to cite lack of time to deliver tobacco treatment as a resource barrier.

Via diffusion of innovations theory, crucial resources should be allocated to settings most suitable for adoption [43]. While both MHCs and SUTCs similarly valued implementation of TFWPs, quantitative data suggest that providers at SUTCs have more time to deliver cessation care. Thus, these settings may be better poised for early adoption to capitalize on limited resources [82]; SUTCs are a potential setting for more in-depth interventions, training, and education [83]. Previous research found that SUTCs are positioned for TFWP implementation when provided with proper support, such as policy development assistance, tobacco education training, treatment resources, and technical assistance [52,55,81,84]. Likewise, tailored brief interventions may be especially relevant for MHCs to offset time as a barrier to cessation provision. Short educational sessions may also substantially increase provider knowledge in MHCs [85]. While provider perceptions of resource availability were similar between MHCs and SUTCs, both healthcare settings are generally low-resourced and could greatly benefit from readily available and low-cost resources. Among these resources are state Quitlines, publicly available online tobacco treatment trainings, free educational materials from tobacco cessation advocacy groups, and organizations that support community healthcare centers with grants for the purchase of NRT.

While qualitative data analysis did not entail comparison of findings based on center type, qualitative findings complemented quantitative results, further contextualizing and explaining their significance. Four main themes emerged that focused on provider- and organizational-level factors that could enable or hinder treating tobacco dependence within these settings. The four themes in connection with quantitative findings revealed a pervasive lack of resources within healthcare settings (e.g., training, institutional resources, policies, patient education materials) and an emergent need to build organizational capacity for TFWP adoption. Many providers were positive about adopting a tobacco-free workplace policy, reporting having the resources to enforce policies and shared salient concerns about secondhand smoke during interviews. Findings indicated relative agreement on the need for tailored interventions that can address the characteristics and organizational capacity of the different types of healthcare centers.

The organizational readiness for change theory informed the analysis and integration of findings, indicating a need to improve organizational preparedness regarding change efficacy and change value for TFWP capacity across healthcare settings, and was applied to produce adapted interventions of program components. The use of RQA allowed the implementation team, in collaboration with partnered centers, to assess needs and tailor plans rapidly and systematically in advance, supporting more deliberate and well-prepared implementation according to each center’s circumstances. The significant disparities and deficits (i.e., fewer tobacco control efforts, limited access to health resources, lack of tobacco-free policy support from local government) experienced by people who use tobacco and live in rural areas emphasize the need for such targeted interventions [86,87]. In interviews, providers shared they felt there was inadequate resource support (i.e., time, community resources) in place at their centers to deliver tobacco treatment, upholding quantitative results on lack of resources to support the provision of cessation services. Lack of time and knowledge on treating tobacco use are documented barriers to provision of smoking cessation support in healthcare settings [88,89].

Integrated findings indicated organizational readiness for having the available resources (i.e., change efficacy) for tobacco-free workplace policy enforcement as well as valuing policy adoption (i.e., change value). Implementation of a tobacco-free policy is an evidence-based intervention [90] that supports tobacco management by increasing awareness of the harms of tobacco use among patients and staff, changes provider attitudes about treating tobacco dependence [82,91], and has been linked to increasing delivery of tobacco treatment interventions in SUTCs [56,92,93]. To further enhance capacity and to build sustainment regarding policy enforcement, centers were provided with quit cards to provide to patients observed smoking on center grounds, guidance on integration and tailoring of a policy to center values, and provision of NRT products. Resources (e.g., quit cards) are designed to facilitate conversation about tobacco cessation between patients and healthcare providers [94].

Providers seemed to perceive the benefits of a tobacco-free workplace policy, but had concerns about staff investment, according to quantitative data. Qualitative data identified two potential contributors to moderate valuation of treating tobacco dependence, low priority and negative provider assumptions about smoking, as necessary to maintain SUD sobriety. Previous research has similarly found a gap in provider perceptions of tobacco as a treatment priority and assumptions about smoking [95,96]. Providers’ intervention practices are influenced by their beliefs about patients, perception of skills, and available referral knowledge [95]. Salient provider attitudes about tobacco use as second (or lower) priority partially depend on the assumption that nicotine addiction is not serious [40]. However, continued evidence of tobacco as a public health priority is prevalent [97,98]. Moreover, tobacco use among people with SUDs causes more deaths than their substance use [99], with over 53% of people with SUDs dying from tobacco-related diseases [100]. Thus, clinical recommendations support the integration of tobacco use disorder interventions into treatment for other substances to promote recovery, enhance treatment outcomes, and reduce harms of ongoing tobacco use [100].

Valuing tobacco treatment as compatible but not a treatment priority coincided with qualitative evidence that providers assumed patient reluctance to quit, that patient smoking was necessary to cope with stress and anxiety, and that smoking is necessary for SUD sobriety. One provider shared their experience with anticipating patient reticence and feeling shocked at patients openly sharing their desire to quit. Provider perceptions of patient desire to quit may motivate their smoking intervention practices; qualitative data support other findings that reveal a link between provider misconceptions and insufficient smoking cessation care delivery [95,101]. Smoking to cope with stress and anxiety has been disproven by evidence that suggests the contrary, that smoking may exacerbate stress and anxiety symptoms [102,103]. Finally, providers emphasized “harm reduction” as a technique through which patients must smoke to maintain sobriety. Harm reduction informs strategies to minimize harmful outcomes from drug use and behaviors associated with drug use [104]. Providers may rationalize the inability to achieve tobacco cessation and treat dependence as harm reduction due to misconceptions that tobacco is a positive, sustainable substitute [105]. Furthermore, smoking to maintain sobriety from other substances has been found to worsen substance use outcomes (i.e., smoking increases use of non-nicotine substances [106]; tobacco use increases relapse to non-nicotine substance use [107]). Instead, research indicates that for patients quitting tobacco use, it enhances their ability to quit other substance use and “maintain sobriety” [106,107,108]. Unfortunately, research suggests that these myriad misconceptions regarding patients’ desire to quit tobacco use and concurrent treatment of tobacco and substance use are a key barrier to providers supporting patients’ quit attempts [57]. Moreover, providers treating SUDs have been found to actively discourage quitting tobacco and non-nicotine substances concurrently, out of fear of jeopardizing SUD recovery or exacerbating mental health conditions [58,108].

In response to study findings, strategies were developed and tailored to program partners’ needs to facilitate TRTTF implementation. Resources were provided for staff to support further provision of tobacco dependence care, based on their needs; i.e., greater or shorter amounts of available time of SUTCS vs. MHCs to devote to tobacco use interventions and trainings. For example, resources and strategies that were adapted based on available time included: (1) providers attended a 45–90 min tobacco education seminar tailored to their setting and populations served; (2) champions were sponsored to attend the Tobacco Treatment Training Program (TTS) at MD Anderson, again according to their capacity and preference (5-day or 1-day prescriber track); and (3) a train-the-trainer program, adjusted to provider capacity that ranged from 30 to 240 min, was implemented. Providers also received training on using brief evidence-based tobacco interventions (e.g., the 5As) [94,109], an evidence-based brief tobacco cessation intervention for provider use at each clinical [55]. Training was also provided on the 5Rs (Relevance, Risks, Rewards, Roadblock, and Repetition) [110] to motivate quit attempts. As partnered centers varied in ability and time to participate in more involved training (i.e., TTS), when time was limited, capacity-building was focused on brief evidence-based tobacco intervention and referral to the Texas Quitline and available resources. Partnered centers were provided with tailored patient education materials for their setting and populations served. Enhancing organizational readiness and building capacity for implementing a TFWP relies on influencing provider practices and supplementing available resources regarding treating tobacco dependence [111]. Providers find tailored education important for patient communication and engagement [112]; this practice is especially important to reduce social disparities in tobacco use [113].

In summary, integrated quantitative and qualitative findings underscore the impact of providers’ attitudes on treating tobacco dependence, and their inextricable reliance on resource availability, and providing tobacco treatment within healthcare settings in rural and/or MUAs treating those with SUDs. Enhancing organizational readiness and building capacity for implementing a TFWP relies on changing provider and organizational values and practices regarding treating tobacco dependence.

### Study Limitations and Implications

The present study provides insight regarding integrating and tailoring interventions for a TFWP in healthcare settings serving rural and MUAs of Texas. As this study focused on the primacy of context for successful program implementation and took place amidst institutional and larger contextual shifts (i.e., COVID-19), findings may not be widely applicable to other similar settings. While it may be possible that COVID-19 affected the generalizability of study findings, as the study period fell within a span of the pandemic, this pandemic has introduced widespread changes in healthcare systems (i.e., telehealth) that persist today and are highly relevant to the rural healthcare settings examined in this study. Impacts from COVID continue to be felt in practice today, particularly in rural settings that substantially adapted their delivery models to remain operational and continue serving their communities during and after the pandemic onset. As such, stratified analyses assessing COVID-era versus post-COVID differences may not be meaningful and are beyond the scope of this study. Additionally, these internal and external contextual factors impacted advancement on program timelines and data collection for our project team, as well as delivery of services, staffing, center closures, and available resources for our program partners. However, this study serves as a model for the application of similar capacity-building efforts and interventions to other settings for assessing and increasing organizational readiness for change to enhance delivery of evidence-based interventions. This study is cross-sectional, and data were collected prior to TRTTF implementation. Longitudinal data and research on provider perceptions of resource availability for and valuation of a TFWP should be further explored to develop an understanding of provider perceptions and interventions for TFWP improvement. For qualitative data collection, to minimize social desirability bias, program critics as well as sympathizers were recruited to represent the study sample more accurately. Overall, findings emphasize the importance of integrating tobacco treatment services into daily care as well as the need to build capacity through provision of resources, training, and policy adoption. Future work may include an evaluative comparison of provider perceptions of available resources for and valuation of a TFWP from pre- to post-implementation across healthcare settings.

This study has methodological strengths. The use of RQA in this study allowed for the perspectives and expertise of providers to guide interventions to build greater organizational capacity for a TFWP. Employing a mixed methods approach allowed researchers and program partners to collaboratively identify organizational needs, implementation barriers and facilitators and develop practical and timely solutions to enhance intervention fit and uptake. Adopting a formative evaluation mixed methods design pre-implementation provided insights into contextual factors that are critical to successful uptake [61]. Quantitative data identified how MHCs and SUTCs are positioned, as well as attitudinal factors and resource needs, regarding implementation of TFWPs. Understanding the comparative preparedness of these healthcare centers for TFWP implementation is critical in planning responsible resource allocation and selection of appropriate implementation strategies to fit the context. Qualitative findings helped elucidate quantitative results, allowing us to build interventions to address identified barriers.

## 5. Conclusions

Findings revealed key insights into MHC and SUTC provider perceptions of resource availability for and valuation of a TFWP. Use of the organizational readiness for change theory aligned with the aim of understanding the multi-level contextual factors that can support or limit uptake of a comprehensive TFWP within these settings. Study findings revealed significant organizational readiness regarding implementation of a tobacco-free workplace policy. However, substantial challenges and limited organizational readiness were found for valuing of and available resources for tobacco treatment provision. Findings indicate that collective behavior or organizational change is needed, which includes changing provider valuation of, and behaviors towards, treating tobacco dependence (i.e., change valence), as well as securing leadership investment to obtain organizational resources to support intervention delivery (i.e., change efficacy). Changes of this order point to a need to change organizational culture.

Qualitative study findings support the conceptual interrelatedness and empirical correlation of the constructs of change valence and change efficacy [46]; for example, participants noted that a lack of training on treating tobacco dependence impeded them from provision of cessation care. This study identified the core implementation components (i.e., the elements or strategies, such as training, needed to drive the implementation of the core intervention components) required by program partners to be successful in implementing the TRTTF program [114]. Strategies will be applied to fill implementation gaps and address center needs to increase the determinants supporting organizational readiness for change. Finally, this study’s mixed methods approach facilitated greater contextual understanding than quantitative or qualitative methods alone and provided clarification regarding specific interventions and capacity building needed for effective future implementation of a TFWP. Potential interventions include provision of tobacco education, training on tobacco cessation interventions (with a focus on training on brief evidence-based interventions for centers with limited time for capacity building), information, and resources to support and streamline referrals, and provision of nicotine replacement treatment, tailored to the needs of program partners. Findings contribute to the development of tailored interventions for healthcare settings interested in implementing TFWPs and addressing tobacco dependence among patients.

## Figures and Tables

**Table 1:** Pre-implementation Characteristics of Medical Healthcare Centers (MHCs) and Substance Use Treatment Centers (SUTCs) Serving Rural and/or Medically Underserved Areas in Texas, US, Agreeing to Participate in a Comprehensive Tobacco-free Workplace Program (*N* = 19).

Select Center Characteristics	All Centers (*N* = 19)		MHCs (*n* = 9)		SUTCs (*n* = 10)		MHCs vs. SUTCs	
Number of…	Mean (SD)	Sum (Range)	Mean (SD)	Sum (Range)	Mean (SD)	Sum (Range)	Statistic	*p*-Value
Counties served	7.89 (11.51)	122 (1–40)	2.56 (1.67)	23 (1–6)	12.70 (14.43)	110 (1–40)	−1.4143	0.1743
Rural or partially rural counties served	7.05 (10.57)	114 (1–33)	2.33 (1.73)	21 (1–6)	11.30 (13.37)	102 (1–33)	−1.2743	0.2188
Counties with medically underserved areas served	5.68 (8.00)	87 (0–29)	2.11 (1.45)	19 (0–5)	8.90 (10.09)	76 (0–29)	−1.3604	0.1905
Unique patients/yearly †	6094.56 (10,688.54)	109,702 (21–46,000)	6102.89 (4255.18)	54,926 (1000–12,139)	6086.22 (14,988.79)	54,776 (21–46,000)	−2.0772	0.0532
Total patient visits/yearly †	21,470.89 (28,909.46)	386476 (21–98,000)	20,127.78 (11,386.81)	181,150 (1750–40,000)	22,814.00 (40,524.87)	205,326 (21–98,000)	−1.5903	0.1302
Staff (inclusive of healthcare providers)	85.68 (136.40)	1628 (3–600)	68.00 (60.31)	612 (15–205)	101.60 (182.71)	1016 (3–600)	0.6940	0.4965
Healthcare providers	61.79 (83.66)	1174 (2–350)	58.67 (52.75)	528 (9–170)	64.60 (107.27)	646 (2–350)	0.8169	0.4247
	**% [*n*]**		**% [*n*]**		**% [*n*]**			
Tobacco-free policy existence	52.63 [10]		44.44 [4]		60.00 [6]		0.4598	0.6563

Note. † Missing information for 1 SUTC; Mann–Whitney tests were conducted to assess differences by center type. Rural counties are defined as those in which all census tracts are rural areas and partially rural counties are those where at least one (but not all) census tract is a rural area [75,76]. Counties with medically underserved areas have at least one geographic area with a shortage of primary care health services or with a shortage of primary care health services for specific populations [77,78,79]. The counties served, rural or partially rural counties served, and counties served with medically underserved areas in the table represent unique counts; stated differently, if more than 1 center served the same county, the county was only counted once in the table. It is important to note that rural/partially rural and medically underserved areas change over time; data reported above were gathered from the Texas State Office of Rural Health, Health Resources and Services Administration in November 2024 [76,79]. SD = standard deviation.

**Table 2:** Differences in Provider (*N* = 347) Perceptions of Existing Resources to Enforce a Tobacco-free Workplace Policy and Resource Barriers to Tobacco Cessation Care Practices in Texas Medical Health Versus Substance Use Treatment Centers.

	All (*N* = 347)	MHC (*n* = 173)	SUTC (*n* = 174)	MHCs vs. SUTCs	
	% Yes [*n*]			Statistic	*p*-Value
**Resource Barriers to Tobacco Treatment**					
Lack of training	65.57 [179]	65.94 [91]	65.19 [88]	−0.108	0.914
Lack of time to deliver care	59.23 [154]	68.38 [93]	49.19 [61]	−2.780	0.006
Lack of reimbursement	42.86 [99]	45.76 [54]	39.82 [45]	−1.176	0.241
Lack of community resources for patient referral	56.30 [143]	63.64 [84]	48.36 [59]	−1.925	0.055
Lack of patient education materials	55.51 [146]	59.26 [80]	51.56 [66]	−1.030	0.304
Existing Resources to Enforce a TFW policy *					
Center has resources	93.08 [323]	91.33 [158]	94.83 [165]	−0.638	0.524

Note. TFW = tobacco-free workplace policy; MHC = medical healthcare center; SUTC = substance use treatment center. Each item was rated on a 5-point Likert scale ranging from “not at all important” to “extremely important.” For analysis, responses were dichotomized as yes (extremely important or very important) or no (moderately important, slightly important, or not at all important). Accordingly, the percentage (%) reported for each item represents the proportion of providers who rated the item as either very important or extremely important. Generalized linear mixed models were used, a nested data structure was considered, and tobacco-free workplace policy existence * at the healthcare center level was controlled. The test statistic and corresponding *p*-value indicate whether the proportion of providers who responded “Yes” differs significantly between MHCs and SUTCs. Results were considered statistically significant when *p* < 0.05. * Refer to Table 1 for % of centers with a tobacco-free workplace policy.

**Table 3:** Differences in Provider (*N* = 347) Perceived Value of Tobacco-Free Workplace Policies and Tobacco Treatment in Texas Medical Health Versus Substance Use Treatment Centers Serving Rural/or Medically Underserved Patients.

	All (*N* = 347)	MHC (*n* = 173)	SUTC (*n* = 174)	MHCs vs. SUTCs	
	% Yes [*n*]			Statistic	*p*-Value
TFW Policy * Value					
TFW policies provide a clean and safe environment for staff and patients	71.76 [249]	72.25 [125]	71.26 [124]	−0.115	0.908
TFW policies may help patients and staff quit smoking	62.25 [216]	63.58 [110]	60.92 [106]	−0.607	0.544
**Tobacco Treatment Value**					
Leadership interest/investment is not a barrier to providing this care	43.72 [101]	42.86 [51]	44.64 [50]	0.135	0.893
Staff interest/investment is not a barrier to providing this care	49.38 [119]	46.03 [58]	53.04 [61]	−0.273	0.785
Cessation care is an important part of the center’s missions	51.01 [170]	55.49 [96]	42.53 [74]	−1.249	0.213

Note. TFW = tobacco-free workplace policy; MHC = medical healthcare center; SUTC = substance use treatment center. Each item was rated on a 5-point Likert scale ranging from “not at all important” to “extremely important.” For analysis, responses were dichotomized as yes (extremely important or very important) or no (moderately important, slightly important, or not at all important). Accordingly, the percentage (%) reported for each item represents the proportion of providers who rated the item as either very important or extremely important. Generalized linear mixed models were used, nested data structure was considered, and tobacco-free workplace policy existence * at the healthcare center level was controlled. The test statistic and corresponding *p*-value indicate whether the proportion of providers who responded “Yes” differs significantly between MHCs and SUTCs. Results were considered statistically significant when *p* < 0.05. * Refer to Table 1 for % of centers with a tobacco-free workplace policy.

**Table 4:** Summary of Qualitative Analysis: Themes and Subthemes Representing Provider Perceptions of Existing Resources for and Perceived Value of Tobacco-Free Workplace Policies and Treating Tobacco Dependence in Texas Medical Health and Substance Use Treatment Centers Servicing Rural and/or Medically Underserved Areas.

Themes	Subthemes	Key Findings
Efficacy Gaps: Additional training and resources are needed to build capacity	(1) Lack of training and knowledge on treating tobacco treating use (2) More resources needed to support tobacco use care efforts	Pervasive lack of standardized and formal training, tobacco use assessments with regular follow-ups, and patient education materials represent a larger need to build capacity to integrate tobacco treatment services
Organizational Factors: Limited institutional frameworks, policies, and practices	(3) Absence of tobacco-free workplace policies (4) Restricted tobacco treatment services	Absence of individual tobacco-free workplace policies across centers is coupled with current tobacco treatment services ranging from no services and lack of awareness regarding the Quitline to some limited services
Valuing: Tobacco treatment was valued as compatible but not a treatment priority	(5) Compatibility with current practices facilitates integration into existing workflows (6) Implementation barriers and facilitators included: constrained staff and leadership investment; treating tobacco is not a treatment priority; extra work for busy staff; low tobacco use rates among staff; high cost of tobacco use; staff willing to implement	Program implementation and training is generally compatible and valued by staff and leadership but treating tobacco use is not their priority
Conflicting Attitudes: Negative assumptions about patient tobacco use and smoking to maintain sobriety	(7) Concerns and misconceptions regarding tobacco use included: Patient hesitance—not interested in quitting or reducing tobacco use; patients need to smoke to cope; concerns about coercion and data collection; harm reduction/smoking to maintain sobriety	Staff emphasize the importance of smoking as a “harm reduction” technique or coping mechanism and express concerns of patient disinterest and reluctance

**Table 5:** Connecting Quantitative and Qualitative Results on Organizational Readiness to Build Intervention Capacity in Texas Medical Health and Substance Use Treatment Centers serving Rural and Medically Underserved Areas of Texas, US.

	Quantitative Results Summary	Qualitative Findings Summary		Integrated Summary of Quantitative and Qualitative Results		Applying Connected Quantitative and Qualitative Results to Inform Capacity Building
**ORC Construct—Change Efficacy:** **Resource Availability**	Resource barriers for providing tobacco cessation services: lack of resources for cessation care provision: training (65.57%; −0.108, *p* = 0.914), time to deliver care (59.23%; −2.780, *p* = 0.006), reimbursement for services (42.86%; −1.176, *p* = 0.214), community resources for patient referral (56.30%; −1.1925, *p* = 0.055), and patient education materials (55.51%; −1.030, *p* = 0.034)	Inadequate supports to deliver cessation care: *“[We’ve been] unsuccessful until we get this training, to be honest with you. We have done nothing…”* *“It can be complicated by the fact that we’re the only provider in our rural area, which means we’re very busy…time is an issue.”* *“More education [materials]. I know our patients need some help; we’re not going the full—we’re not doing everything we can.”*		Findings from both components indicate lack of adequate resources and need for organizational capacity building to support effective implementation of the TRTTF program		Resources provided to support partners included: (a) all-staff tobacco education training tailored to SUTC or MCH setting and patients served; (b) PCs sponsored to attend TTS training to gain expertise in treating tobacco use; (c) brief tobacco use interventions training (5A’s, 5R’s); (d) guidance to facilitate direct TQ referral; (e) selection or development of patient education materials tailored to their settings and populations such as veterans, IDD populations; (f) setting tailored T-t-T program
	Available resources for task demands, to enforce TFW policy: providers reported having the resources to enforce TFWP (93.08%; −0.638, *p* = 0.524)	Limited concern about TFW policy adoption and enforcement: *“I really don’t see that we’re going to have any issues staying a tobacco-free workplace. I think everybody is invested in this. We all see that as a recovery-oriented agency that this is something that we really need to put forth. We need the public to see that we’re invested in this as well.”*		Both quantitative and qualitative results indicate capacity in change efficacy for TFW policy adoption and enforcement	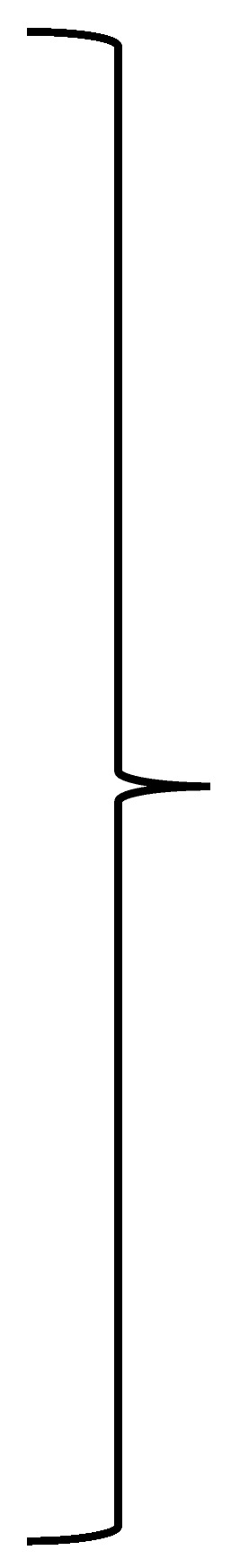	To enhance capacity, team offered partners: (a) Quit cards for patients seen smoking with TQ contact information; (b) guidance on developing TFW policy aligned with center values; (c) endorsed provision of NRT products and tobacco services to staff
**ORC Construct—Change Valence**: **Value placed on proposed change**	TFW policy value: satisfactory endorsement of TFW policy benefits on: provides patients and staff clean and safe space (71.76%; −0.115, *p* = 0.908); may help to quit smoking (62.25%; −0.607 *p* = 0.544)	Providers support TFW policy adoption; concerns about second-hand smoke: *“During breaks…a lot of [patients] smoke, and then it would smell very badly. I always prefer to avoid it when I can because it can affect my breathing.”*		Results from both components indicate organizational capacity in change value for TFW policy adoption and enforcement		
	Cessation Care Value: tepid support from leadership (43.72%; 0.135, *p* = 0.893) and staff (49.38%; −0.273, *p* = 0.785) on investing in cessation interventions	Concerns about patient loss of census/complaints: *“Possibly [due to] lack of training. We usually don’t implement things here that are not supported by upper administration so that clients could come back and complain.”*	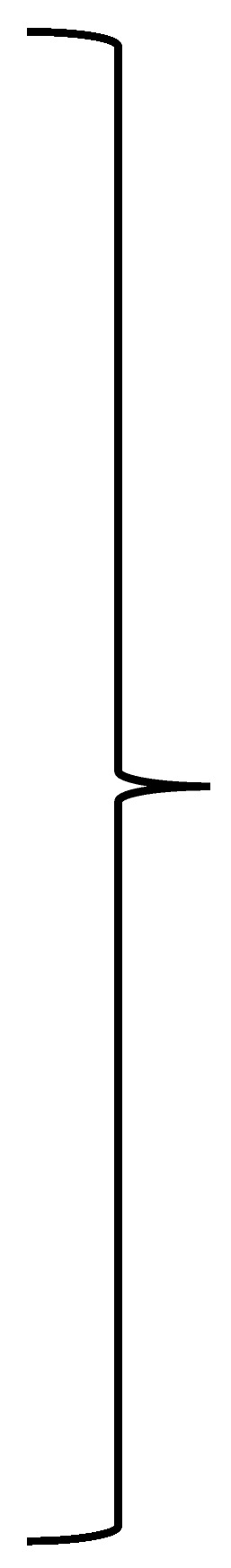	Qualitative findings support quantitative results that change valence for investing in and delivering patient tobacco cessation care is only moderately valued by leadership and staff	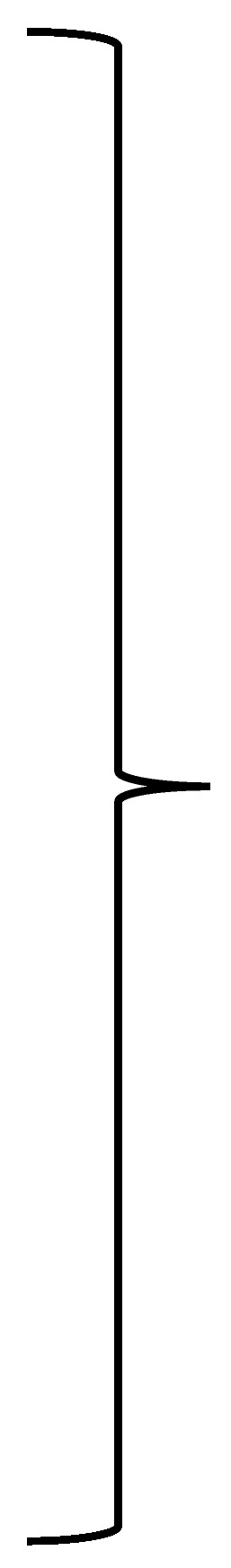	To increase valuing of treating tobacco use, center-wide training tailored to MHC/SUTC partners on: (a) how tobacco cessation bolsters SUDs and mental health recovery, how tobacco use is linked to ACEs, calling for the integration of TIC, harm of ENDs use, (MHCs/SUTCs); adverse effects on diabetes, HIV, eye and dental health, CVD, COPD, asthma, and pregnancy (MHCs); (b) dispelling misconceptions and fears such as TFW policy adoption is not linked to decreased patient census; (c) specific topics requested included articles, videos on treating ENDs among youth and during pregnancy
	Relative Valuing of Cessation Interventions: about half (51.01%; −1.249, *p* = 0.213) of providers perceived cessation care as central to their center’s mission	Contending patient priorities: *“…our main concern when they come in is the addiction of alcoholism or substance abuse, right?… because we have so many different issues that we deal with in relation to the population we serve. So, it [tobacco use] is a concern, but it’s not a top priority.”*				

Note: ORC = Organizational Readiness for Change, TRTTF = Taking Rural Texas Tobacco Free; SUTCs = substance use treatment centers; MHCs = medical health centers; PCs = program champions; TTS = tobacco treatment specialist; T-t-T = train-the-trainer; TQ = Tobacco Quitline; IDD = intellectual and developmental disabilities; NRT = nicotine replacement therapy; SUDs = substance use disorders; ACEs = adverse childhood experiences; TIC = trauma-informed care; HIV = human immunodeficiency virus; CVD = cardiovascular disease; COPD = chronic obstructive pulmonary disease; ENDs = electronic nicotine devices; TFW = tobacco-free workplace; TFWP = tobacco-free workplace program.

## Data Availability

Data are not publicly available due to privacy restrictions. The data that support the findings of this study are available from the senior author, L.R.R., upon reasonable request.

## Supplementary Materials

The following supporting information can be downloaded at: https://www.mdpi.com/article/10.3390/ijerph23070898/s1, File S1: Leader Readiness; File S2: Pre-Implementation Survey; File S3: Pre-Staff Interview Guide.
