# Supplementary material for: Provider Perspectives on a Tobacco-Free Workplace Program in Healthcare Settings Serving Rural and Medically Underserved Areas of Texas: A Mixed Methods Study on Perceived Resource Availability and Value
Source: Int J Environ Res Public Health. 2026 Jul 12;23(7):898. doi: 10.3390/ijerph23070898 (PMC13411692; doi:10.3390/ijerph23070898)
Supplement: Supplementary file 1 [file ijerph-23-00898-s001.zip › File S1-Leader Readiness.pdf]

Taking *Rural* Texas Tobacco Free

CEO/Leader Needs and Readiness Survey

Purpose of Project

The University of Texas MD Anderson Cancer Center, the University of Houston, and Integral Care (IC) have received a grant from the Cancer Prevention & Research Institute of Texas (CPRIT) to disseminate *Taking Rural Texas Tobacco Free*, a multi-component tobacco-free workplace program to selected Substance Use Treatment Centers (SUTCs) and Health Centers (e.g., Federally Qualified Health Centers [FQHCs]) located within or serving rural and medically underserved areas of Texas. This award offers a tremendous opportunity to implement a comprehensive evidence-based tobacco-free workplace program that includes education, screening, treatment, and outreach components in addition to a tobacco-free workplace policy at your center/centers.

Through our grant (PP210003), *Taking Rural Texas Tobacco Free* will provide training/education, consultation, policy development and implementation assistance, treatment resources, and practical guidance, as needed, to implement and/or sustain the changes that will result in the long-term prevention of tobacco-related cancers among your patients, employees, and the larger communities in which they live. Increasing tobacco cessation rates is critically important to the advancement of cancer prevention in Texas, and we are glad you have decided to join us in achieving this important goal of improving the health of the Texans we employ and serve.

Are you already screening patients for tobacco use? Already have a tobacco-free workplace policy? No problem! We meet centers “where they are” in the use of evidence-based interventions for tobacco control and cessation. However, for centers that need it, we will offer money for tobacco-free workplace signage, sponsor selected employees to attend a Certified Tobacco Treatment Specialist training at MD Anderson Cancer Center, provide a starter kit of Nicotine Replacement Therapies for patients and employees, general and specialized training to providers (e.g., Motivational Interviewing training), health promotion information (e.g., rack cards, posters, flyers) for dissemination to patients, and a host of suggestions for how to sustain the program beyond the implementation period. Depending on your needs and characteristics (e.g., size) as a center, the active implementation period ranges between 6-12 months, on average. Evaluation processes may span beyond this time.

This survey is being sent to you because we have a completed Memorandum of Understanding on file for your center (which you can preview here if you are not familiar). This survey assesses the needs and readiness of each center regarding the implementation of the Program. Following your completion of this survey, we will be in touch with you over the next week or so for next steps and to clarify any questions we may have about your responses. We will also be available to answer any questions you may have.

Procedures

If you agree to complete this survey, you will be asked about your center's characteristics, readiness and needs regarding tobacco control interventions. Based on the questions asked, you may wish to have your last annual report data (e.g., about patients served) handy to be able to respond with accuracy. The survey should take between 10 to 15 minutes to complete. Your responses will not be shared with your employees or other centers.

Taking Rural Texas Tobacco Free will be actively implemented in at least 18 SUTCs and 10 FQHCs located within or serving rural and medically underserved areas of Texas over a 3 year period. We will select center participants on a first-come, first-served basis, but are willing to work with you to select an internal implementation timeline that works best for your center. Survey data will be maintained for at least three years following the completion of the program aims.

By completing the survey, you acknowledge that you: (a) have read the goal of the project, (b) have decided to complete the part of the project described here, and (c) understand that you are free to refuse further participation at any time.

During the course of this program implementation, the project team at The University of Texas MD Anderson Cancer Center, The University of Houston, and Integral Care will be collecting information about your center that they may share with other parties that include health authorities, project monitors who check the accuracy of the information, and individuals who would put all the project information together in report form. By answering the questions, you are providing authorization for the project team to use and share your center's de-identified information (i.e., recipients receive aggregate information that describe all participating centers and do not include specific center's information) at any time with the above-mentioned entities/personnel. Please note that your decision to participate (or not) will not be shared with your employer. Should you choose to participate, your individual responses to this survey will not be shared with your employer. If you do not want to authorize the use and disclosure of this information, you should choose not to answer these questions. You may contact Dr. Lorraine Reitzel, the Project Director, at 346-834-8095 with questions. This project has been reviewed by the University of Houston Committee for the Protection of Human Subjects (713-743-9204) and The University of Texas MD Anderson Cancer Center's Quality Improvement Assessment Board.

Any questions regarding your rights as a research subject may be addressed to the University of Houston Committee for the Protection of Human Subjects (713-743-9204).

[include CAPTCHA]

[include "click here if you consent to participate and to complete this survey"]

Draft survey (* = forced to answer)

1. What is your name? *

2. Please confirm a good email address where we can reach you. *

3. What is the name of your center? *

4. How many physical locations/clinics does your center have? *

5. How many physical locations/clinics does your center have that are located in (or that serve individuals from) rural/medically underserved areas of Texas? *

Below, we will ask you questions that specifically pertain to 1 or more of your clinics that might be interested in participation in our program. We are interested in your **answers with regard to these clinics only.** Based upon the focus of our grant, at least some of these clinics must be located within rural or medically underserved areas of the state OR must serve patients from rural or medically underserved areas of the state.

6. How many of your clinics would consider participating in our program? *

   Please provide the information for clinics which would consider participating in our program. If there are more than 15 clinics, please specify the 15 that you would like to prioritize and please discuss additional clinic needs/interest with TTTF staff.

	Name of Clinic	Physical Address
Clinic 1		
Clinic 2		
Clinic 3		
Clinic 4		
Clinic 5		
Clinic 6		
Clinic 7		
Clinic 8		
Clinic 9		
Clinic 10		
Clinic 11		
Clinic 12		
Clinic 13		
Clinic 14		
Clinic 15		

7. What county/counties do the clinics you refer to in the above question serve? (please choose all that apply) *

8. Considering all of your potentially participating clinics globally as "your center," what proportion of your center's patients come from each of the following county/counties? It's ok if your estimates are not exact, but please try to be as precise as possible*

9. What is the number of <u>unique</u> patients aged 16 years or older your center served last year? It's ok if your estimates are not exact, we just want a general idea. Please count both residential and outpatient patients, as applicable. (text box answer, formatted for number entry only) *

10. What is the number of <u>total</u> patient contacts your center had last year (please estimate this for patients aged 16 years or older)? It's ok if your estimates are not exact, we just want a general idea. Please count both residential and outpatient patients, as applicable. (text box answer, formatted for number entry only) *

11. What percentage of your patients aged 16 or older are engaged in the criminal justice system in some way (e.g., on parole or probation, jail diversion, alternative to jail, etc.)? It's ok if your estimates are not exact, we just want a general idea. If you collect this information but need help pulling a report on it, please reach out to our team for assistance. (Answer options = sliding ruler; plus "We do not collect this information/I cannot estimate this.")

12. What percentage of your patients aged 16 or older are: It's ok if your estimates are not exact, we just want a general idea. If you collect this information but need help pulling a report on it, please reach out to our team for assistance.
    _____ % men
    _____% women
    _____% non-binary
    _____% unknown/ We do not collect this information/I cannot estimate this.

13. What percentage of your patients aged 16 or older… It's ok if your estimates are not exact, we just want a general idea. If you collect this information but need help pulling a report on it, please reach out to our team for assistance.
    … have been homeless in the past 5 years or are high risk for future homelessness? _____ %
    … identify as sexual minorities? _____ %
    … are pregnant? _____ %
    … are within 6 months postpartum? _____%

(Answer options = sliding ruler; plus "We do not collect this information /I cannot estimate this.")

14. What percentage of your patients aged 16 or older have a diagnosis of the following substance use disorders… It's ok if your estimates are not exact, we just want a general idea. If you collect this information but need help pulling a report on it, please reach out to our team for assistance.
    … Alcohol _____ %
    … Cannabis _____ %
    … Stimulant _____ %
    … Hallucinogen _____ %
    … Opioid _____ %
    (Answer options = sliding ruler; plus "We do not collect this information /I cannot estimate this.")

15. What percentage of your patients aged 16 or older have a diagnosis of one of the following mental illnesses… It's ok if your estimates are not exact, we just want a general idea. If you collect this information but need help pulling a report on it, please reach out to our team for assistance.
    … Depression _____ %
    … Trauma and Stress Related Disorder(s) (eg., PTSD) _____ %
    … Anxiety Disorder(s) (eg., GAD, Phobias) _____ %
    … Personality Disorder(s) (eg., Borderline, Antisocial) _____ %
    … Other serious illness(es) (eg., Bipolar Disorder, Schizophrenia) _____ %
    (Answer options = sliding ruler; plus "We do not collect this information /I cannot estimate this.")

16. What percent of your patients aged 16 years or older smoke conventional cigarettes? It's ok if your estimates are not exact, we just want a general idea. If you collect this information but need help pulling a report on it, please reach out to our team for assistance. (Answer options = sliding ruler; plus "We do not collect this information."*

17. What percent of your patients aged 16 years or older use other (non-cigarette) tobacco products (including but not limited to e-cigarettes and vaping products)? It's ok if your estimates are not exact, we just want a general idea. If you collect this information but need help pulling a report on it, please reach out to our team for assistance. (Answer options = sliding ruler; plus "We do not collect this information/I cannot estimate this.")*.

18. Does your center mandate that every client aged 16 years or older is screened for tobacco use <u>at intake</u> and that this is documented in the client record?
    a. Yes

   b. No
   c. I do not know

19. Does your center mandate that every client aged 16 years or older who indicates tobacco use at intake is given a comprehensive Tobacco Use Assessment (includes information like patient's smoking status, smoking history, cigarettes, or packs smoked per day, number of years smoked, number of years since quitting smoking, etc.)
    a. Yesho
    b. No
    c. I do not know

20. Does your center mandate that every client aged 16 years or older is screened for tobacco use at each post-intake appointment and that this is documented in the client record?
    a. Yes
    b. No
    c. I do not know

21. How are assessments for tobacco dependence documented?

    ☐a. They are a mandatory part of the patient's written/paper record.

    ☐b. They are a mandatory part of the patient's electronic health record.

    ☐b. They are included at the provider's discretion.

    ☐c. They are not documented in any regular fashion.

    d. I do not know how tobacco dependence assessments are documented.

22. Does your center have at least 1 professional on site who can prescribe tobacco cessation medications (e.g., Chantix, Zyban)?
    a. Yes
    b. No
    c. I do not know

23. Does your center have an on-site pharmacy?
    a. Yes
    b. No
    c. I do not know

24. What is your number of employees across locations participating in this project? If certain departments/divisions (e.g., emergency room, inpatient detox) are participating and not others, please only count employees from participating departments/divisions. It's ok if your estimates are not exact, we just want a general idea. Full time employees: Non-full time employees (Part-time employees, PRN [as-needed], travel, etc.): (text boxes to answer, formatted for number entry only)*

25. How many of your employees have direct patient contact? Employees with the following credentials (but not only these credentials) are typically those who have direct patient contact: NP, LVN, RN, APN, CNA, MA, QMHP, MD, LCDC, LSW, etc. Employees might also have titles like recovery coach, patient navigator, or peer support specialist. It's ok if your estimates are not exact, we just want a general idea. Full time employees: Non-full time employees (Part-time employees, PRN [as-needed], travel, etc.): (text boxes to answer, formatted for number entry only)*

26. What percent of your employees currently use tobacco? It's ok if your estimates are not exact, we just want a general idea. If you collect this information but need help pulling a report on it, please reach out to our team for assistance. (Answer options = sliding ruler; plus "We do not collect this information/I cannot estimate this.").*

27. At what stage is your center in implementing a tobacco-free workplace policy? (select all that apply)

☐a. We have implemented a tobacco-free workplace policy and it is going well.

☐b. We have implemented a tobacco-free workplace policy but we could use help with quality assurance/monitoring and remedying violations.

☐c. We have approved a tobacco-free workplace policy, but it has not yet been implemented.

☐d. We have a tobacco-free workplace policy that allows smoking in designated areas on the property.

e. We are interested in implementing a tobacco-free workplace policy.

☐f. We are exploring options concerning a tobacco-free workplace policy.

☐g. We do not plan to adopt/implement a tobacco-free workplace policy under any circumstances.

h. Please explain here if nothing above "fits" or if your various locations /clinics have different practices/policies. (text box)

28. What services does your center currently provide to help people quit using tobacco? (check all that apply)
    a. We do not provide tobacco cessation services or resources.
    b. We refer patients to Texas Tobacco QuitLine or other services/groups/parties.
    c. We provide individual and/or group counseling for tobacco cessation.
    d. We provide nicotine replacement therapy (NRT).
    e. We provide varenicline (Chantix) and/or bupropion (Wellbutrin/Zyban).
    f. We provide patients with health promotion materials on tobacco use and quitting tobacco.
    g. We use Motivational Interviewing to build internal patient motivation to quit.
    h. Please explain here what else you may do if not in the list above and/or if your various locations /clinics have differing practices/policies. (text box)

29. Currently, how would you rate the capacity of your center to collect tobacco-related data; for example, # of patients a month screened for tobacco use, # of patients provided cessation assistance; # of patients a month who made a quit attempt, # of patients a month who successfully quit tobacco? (0 to 10 scale where 0 = very low, 5 = average, and 10 = very high)

30. Currently, how would you rate the capacity of your center to use tobacco-related data to tailor or target interventions to patient groups or special populations that you serve? (0 to 10 scale where 0 = very low, 5 = average, and 10 = very high)

Please rate your level of agreement with the items below, which refer to the implementation of *Taking Rural Texas Tobacco Free* at your center, which would require the adoption of a tobacco-free workplace policy (no designated smoking areas), practices around screening and treating tobacco use among patients at every contact, providing nicotine replacement therapy to patients and employees, etc., using the guidance, training, and resources we would provide. More about the obligations for participation can be found in the Memorandum of Understanding, which you can access here. Although you may not know how all people in your center feel, please just do your best to estimate what you think is the most representative answer for each item below. Remember, **please answer regarding 1 or more of your clinics that are located in rural/medically underserved areas of Texas**:

31. People who work here feel confident that the center can get people invested in implementing this change.

☐1. Disagree

☐2. Somewhat disagree

☐3. Neither agree nor disagree

☐4. Somewhat agree

☐5. Agree

32. People who work here are committed to implementing this change.

☐1. Disagree

☐2. Somewhat disagree

☐3. Neither agree nor disagree

☐4. Somewhat agree

☐5. Agree

33. People who work here feel confident that they can keep track of progress in implementing this change.

☐1. Disagree

☐2. Somewhat disagree

☐3. Neither agree nor disagree

☐4. Somewhat agree

☐5. Agree

34. People who work here will do whatever it takes to implement this change.

☐1. Disagree

☐2. Somewhat disagree

☐3. Neither agree nor disagree

☐4. Somewhat agree

☐5. Agree

35. People who work here feel confident that the center can support people as they adjust to this change.

☐1. Disagree

☐2. Somewhat disagree

☐3. Neither agree nor disagree

☐4. Somewhat agree

☐5. Agree

36. People who work here want to implement this change.

☐1. Disagree

☐2. Somewhat disagree

☐3. Neither agree nor disagree

☐4. Somewhat agree

☐5. Agree

37. People who work here feel confident that they can keep the momentum going in implementing this change.

☐1. Disagree

☐2. Somewhat disagree

☐3. Neither agree nor disagree

☐4. Somewhat agree

☐5. Agree

38. People who work here feel confident that they can handle the challenges that might arise in implementing this change.

☐1. Disagree

☐2. Somewhat disagree

☐3. Neither agree nor disagree

☐4. Somewhat agree

☐5. Agree

39. People who work here are determined to implement this change.

☐1. Disagree

☐2. Somewhat disagree

☐3. Neither agree nor disagree

☐4. Somewhat agree

☐5. Agree

40. People who work here feel confident that they can coordinate tasks so that implementation goes smoothly.

☐1. Disagree

☐2. Somewhat disagree

☐3. Neither agree nor disagree

☐4. Somewhat agree

☐5. Agree

41. People who work here are motivated to implement this change.

☐1. Disagree

☐2. Somewhat disagree

☐3. Neither agree nor disagree

☐4. Somewhat agree

☐5. Agree

42. People who work here feel confident that they can manage the politics of implementing this change.

☐1. Disagree

☐2. Somewhat disagree

☐3. Neither agree nor disagree

☐4. Somewhat agree

☐5. Agree

43. We know how much time it will take to implement this change.

☐1. Disagree

☐2. Somewhat disagree

☐3. Neither agree nor disagree

☐4. Somewhat agree

☐5. Agree

44. We know what resources we need to implement this change.

☐1. Disagree

☐2. Somewhat disagree

☐3. Neither agree nor disagree

☐4. Somewhat agree

☐5. Agree

45. We know what each of us has to do to implement this change.

☐1. Disagree

☐2. Somewhat disagree

☐3. Neither agree nor disagree

☐4. Somewhat agree

☐5. Agree

46. We have the expertise we need to implement this change.

☐1. Disagree

☐2. Somewhat disagree

☐3. Neither agree nor disagree

☐4. Somewhat agree

☐5. Agree

47. We have the time we need to implement this change.

☐1. Disagree

☐2. Somewhat disagree

☐3. Neither agree nor disagree

☐4. Somewhat agree

☐5. Agree

48. We have the skills to implement this change.

☐1. Disagree

☐2. Somewhat disagree

☐3. Neither agree nor disagree

☐4. Somewhat agree

☐5. Agree

49. We have the resources we need to implement this change.

☐1. Disagree

☐2. Somewhat disagree

☐3. Neither agree nor disagree

☐4. Somewhat agree

☐5. Agree

50. The timing is good for implementing this change now.

☐1. Disagree

☐2. Somewhat disagree

☐3. Neither agree nor disagree

☐4. Somewhat agree

☐5. Agree

51. The timing will be good for implementing this change next year.

☐1. Disagree

☐2. Somewhat disagree

☐3. Neither agree nor disagree

☐4. Somewhat agree

☐5. Agree

52. We believe that implementing this change is a good idea.

☐1. Disagree

☐2. Somewhat disagree

☐3. Neither agree nor disagree

☐4. Somewhat agree

☐5. Agree

53. We value this change.

☐1. Disagree

☐2. Somewhat disagree

☐3. Neither agree nor disagree

☐4. Somewhat agree

☐5. Agree

54. We believe this change will make things better.

☐1. Disagree

☐2. Somewhat disagree

☐3. Neither agree nor disagree

☐4. Somewhat agree

☐5. Agree

55. We believe this change will benefit our community.

☐1. Disagree

☐2. Somewhat disagree

☐3. Neither agree nor disagree

☐4. Somewhat agree

☐5. Agree

56. We feel this change is compatible with our values.

☐1. Disagree

☐2. Somewhat disagree

☐3. Neither agree nor disagree

☐4. Somewhat agree

☐5. Agree

57. Are you interested in working with *Taking Rural Texas Tobacco Free* to implement this program at your center?

    a. yes

b. no
c. I am unsure and need more information

58. Please comment on special assistance you might anticipate needing during program implementation:

This is the final page of the survey. You can use the forward arrow to submit your response. Otherwise, you can use the back arrow to review any of your responses.

Thank you for completing this survey. We will be in touch with you after we review your responses with regard to your interest in working with us!
