# Supplementary material for: Provider Perspectives on a Tobacco-Free Workplace Program in Healthcare Settings Serving Rural and Medically Underserved Areas of Texas: A Mixed Methods Study on Perceived Resource Availability and Value
Source: Int J Environ Res Public Health. 2026 Jul 12;23(7):898. doi: 10.3390/ijerph23070898 (PMC13411692; doi:10.3390/ijerph23070898)
Supplement: Supplementary file 1 [file ijerph-23-00898-s001.zip › File S3-Pre-Staff Interview Guide.pdf]

Taking Texas Tobacco Free among Substance Users within Community-based Healthcare Settings in Rural and Medically Underserved Areas Across Texas

Pre-implementation Interview Guide for ONLINE/In-Person Individual or Group Interview (as preferred by participants) for Staff

Probing questions: *Questions to be asked to help the interviewee to elaborate, in the event that additional information is not provided spontaneously.*

Interviewer prep work: *Review Partnership Agreement to determine the program components being implemented at each center prior to interview.*

Introduction: I'm Isabel Leal from The University of Texas MD Anderson Cancer Center [and/or other research project personnel name here], I'm an Assistant Professor/[insert position of other research personnel], with the Taking Texas Tobacco Free (TTTF) program that provides support for disseminating and implementing a tobacco-free workplace program through our collaboration with colleagues from the University of Houston and is funded by the Cancer Prevention and Research Institute of Texas.

Thank you so much for agreeing to participate in this interview. We are interested today in talking with you and exploring your ideas about how we can be most effective in assisting you in implementing a comprehensive tobacco-free workplace program into your setting. As part of this research, we will be meeting with up to 250 stakeholders from substance use treatment centers and health centers who are participating in this program to gather similar information.

We anticipate that this interview/focus group will take about 24-40 [individual] or 45-60 minutes [group] of your time and will be conducted either in-person or virtually online – according to your health center's preferences. Your participation in this interview is voluntary. If you don't feel comfortable answering a question or want to stop, you can stop at any time. You are welcome to refuse to participate or refuse to answer any and all questions without penalty or loss of benefits to which you are otherwise entitled. We do not anticipate any significant risks associated with participation in this interview. The potential benefit of your participation is that your feedback will help us to adapt the program to the needs of your specific center and assist the clients you serve in quitting tobacco use, thus preventing tobacco-related cancers.

The information that you provide us with will only be used to help us assist you in implementing this tobacco-free program into your setting. Although we will combine your responses with interviews/focus groups from other participating centers/programs, we would like the ability to use selected quotes from today in our future academic work, and/or grant funding applications and reports. We will not use your name, only deidentified data will be reported and personal identifiers will be removed from the information you provide; we will use a made-up name instead. You will be compensated with a $20 Amazon gift card at the end of this interview in appreciation of your time and effort. Also, you can contact me by email or phone at any time if you have concerns about the program of your participation.

[provide business card in-person or Chat]

We would like to audio-record [in-person] / audio- and video-record [virtual] this meeting so that we make sure to accurately capture your feedback. Within a month after this meeting, a professional company with strict privacy and confidentiality procedures will write down everything that has been said here and the recording will be destroyed. Again, your personal information, such as your name, will

not be included in any written records. Do you agree to be audio-recorded [in-person] / audio- and video-recorded [virtual]?

[Wait for participant(s) agreement or not to be recorded]

[Participant did not agree to be recorded]

We will not audio-record [in-person] / audio- and video-record [virtual] this meeting. Instead, [I/name of note taker] will take notes. We will not include any personal information such as your name or where you work in our written records.

[Group interviews only] We ask that all participants respect each other's privacy and not repeat any information shared here today, but we cannot promise that everyone will.

So that I can accurately identify who is speaking when I transcribe this interview, I ask that you identify yourself before you speak by stating your first name or initials. Is that alright with you?

What questions, if any, do you have about this? May we get started?

We'll start with a brief description of the TTTF Program components that your center will be adopting to assist clients and staff to quit using tobacco: [Brief description/reminder of the program components of The TTTF Program being implemented for the center/program being interviewed]: 1) adoption of a tobacco-free workplace policy; 2) regular tobacco use screenings of the people you serve; 3) train providers on provision of evidence-based tobacco use interventions and culturally and trauma-informed care; 4) provision of resources – a) ongoing implementation guidance from team and b) resources, which includes starter Nicotine Replacement Therapy (NRT), tobacco-free signage, and dissemination materials)

1) Please state your name, employer, and position.

[share passive dissemination materials]

<u>Adaptation/Dissemination materials</u>

2) What do you think about our dissemination materials? How useful will they be in your setting? What would you recommend changing to better suit your facility? (Probes)
    a. What other special populations, languages, or age groups, etc., would you like to see included?

<u>Current screening practices</u>

3) What is included in the tobacco screenings of clients, if any, conducted by your center – i.e., other tobacco products (smokeless tobacco, e-cigarettes?)(Probes)
    a. Do you ask about other substance use – i.e., drug and alcohol use?
    b. If your center does not conduct tobacco screenings, why not?
    c. If so, how often? Who conducts these screenings?
    d. How are these screenings documented? (EHR?)

<u>Tobacco-free workplace policies</u>

4) What tobacco-free policies or restrictions do you currently have in place for indoors (vaping) and outdoors in your center?

Supports for tobacco use assessments and treatment

5) What kind of tobacco education trainings have you received, either through your center or another organization? (Probes)
    a. Who receives these trainings on tobacco education, and how often?
    b. What is covered in these trainings?
        i. General harms of tobacco use?
        ii. How to treat tobacco use generally or in special populations?
    c. How long do these trainings generally last and who provides them?
    d. What do you think should be included in these trainings? E.g., e-cigarettes, secondhand smoke, smoking while pregnant?

Services provided and potential for improvement

6) What kinds of tobacco cessation services do you currently provide to clients and staff at your center? (nicotine replacement therapy, individual/group counseling) Probes)
    a. What do you currently do to help your clients who want to quit smoking?
        i. If clients are referred out, what is the referral process – direct, or passively given the Quitline number to call on their own?
    b. Are there ways in which you could integrate smoking cessation with other services that you provide? (For example, do you think clients' groups could discuss smoking cessation?)
    c. Would you be able to dedicate staff to providing smoking cessation care?
    d. If you had a chance to create a smoking cessation program for your clients, what would you like to see in your smoking cessation program? What would really help clients quit smoking?

Program compatibility

7) How well do you think the intervention fits with existing work processes and practices in your setting?
    a. What are likely issues or complications that may arise?

Individual concerns about program

8) How do you honestly feel about the intervention being used in your facility?
    a. How do you feel about the plan to implement the intervention in your facility?
    b. Would you say you have feelings of anticipation? Stress? Enthusiasm? Why?

Program implementation barriers and facilitators

9) a. [For centers that have already adopted a tobacco-free policy] What are some things that have made it hard to sustain your center's tobacco-free workplace policy, and what is needed to overcome these barriers in terms of (Probes): (If needed, prompt: On the level of organizational resources, clinicians, clients?)

[For centers that have not yet adopted a tobacco-free policy] What do you foresee will be the main barriers to adopting a tobacco-free workplace policy in your center, and what is needed to overcome these barriers in terms of (Probes):

b. [For centers that have already adopted a tobacco-free policy] Likewise, what have been the main facilitators to delivering and sustaining your center's tobacco-free workplace policy? How do you think these facilitators can be enhanced/supported regarding (Probes):

(If needed, prompt: On the level of organizational resources, clinicians, clients?)

i) Does your center have any policies regarding treating clients that might support or hinder tobacco control efforts in your center?

[For centers that have not yet adopted a tobacco-free policy] Likewise, what do you foresee will be the main facilitators to adopting a tobacco-free workplace policy in your center? And how do you think these facilitators can be enhanced or supported? (Probes, above):

Organizational attitudes towards treating tobacco use

10) Can you describe the general attitude towards treating tobacco at your center? (Probes)
    a. Is addressing tobacco dependence among your clients considered a treatment priority for clinicians? If not, why not?
    b. Is addressing tobacco dependence among your clients supported or considered a treatment priority by leadership? Why or why not?
    c. Would you describe your center's efforts to address tobacco as successful or unsuccessful, and why?

Additional information

11) Is there anything else that you would like to share about how we can make this project run smoothly in your center that we have not covered?

Closing: Thank you so much for your time and for sharing your thoughts today.
